# Case report: Efgartigimod combined with intravenous methylprednisolone in a case of co-occurrence of unexplained myasthenia gravis, inflammatory myopathy, and fulminant myocarditis

**DOI:** 10.3389/fimmu.2026.1769065

**Published:** 2026-04-01

**Authors:** Hongxia Yang, Zulin Pan, Ying Yang, Yaxuan Wang, Biqi Cheng, Guoyan Qi

**Affiliations:** Center of Treatment of Myasthenia Gravis, Shijiazhuang People’s Hospital, Shijiazhuang, China

**Keywords:** efgartigimod, fulminant myocarditis, inflammatory myopathy, intravenous methylprednisolone, Myasthenia gravis

## Abstract

The co-occurrence of myasthenia gravis (MG), inflammatory myopathy (IM), and fulminant myocarditis (FM) has rarely been reported. The few previous articles were mostly case reports or small sample data analysis, and the etiology was mostly attributed to thymoma and/or the application of immune checkpoint inhibitors (ICIs). We report a 74-year-old woman with an acute onset of general weakness, chest tightness, shortness of breath, dysarthria, masticatory weakness and dysphagia, without myalgia or rash. Acetylcholine receptor antibody was positive. Serum creatine kinase, transaminase, and myoglobin were higher than normal. A variety of autoimmune antibodies including anti-Ro-52, JO-1, nRNP and dsDNA antibodies were positive. Chest CT showed no thymoma, bronchiectasis with infection in both lungs. Some of them showed interstitial changes. Electromyography repetitive nerve stimulation of the bilateral deltoid muscle demonstrated a decremental response at low frequencies without an incremental response at high frequencies. Needle electromyography revealed spontaneous potentials. Echocardiography showed that left ventricular wall motion was generally reduced, left ventricular systolic function was reduced, and cardiac function was EF18%. Muscle biopsy suggested myogenic injury. The patient was diagnosed with MG-IM-FM coexisting without thymoma and without ICIs. After treatment with Efgartigimod combined with intravenous methylprednisolone, the symptoms were relieved, serum creatine kinase, myoglobin and other laboratory indicators and cardiac function returned to normal. MG-IM-FM is a complex disease with rapid onset, rapid progression and critical condition. Clinicians should be on high alert and early intervention is the key to improve the prognosis. The immune mechanism of its occurrence is still unclear and needs to be further studied. Traditional glucocorticoid is still the first-line drug, but the new targeted drug Efgartigimod has less adverse reactions and faster onset of action, which may become a good choice, but needs to be verified by a larger sample.

## Introduction

Myasthenia gravis (MG) is an autoimmune neuromuscular junction disease mediated by antibodies such as acetylcholine receptors (AChR), characterized primarily by muscle weakness and easy fatigability (1). Recent studies have found that MG can coexist with other autoimmune diseases (2), although its coexistence with inflammatory myopathy (IM) is rare, it has unique clinical significance. Literature reports indicate that it can manifest as myositis ranging from subclinical to diffuse muscle involvement, particularly characterized by weakness of the distal upper limbs (3). This comorbidity may be related to muscle inflammation or a common immune attack on the neuromuscular junction and muscle tissue (4–6). Notably, some MG-IM patients have no history of thymoma or immune checkpoint inhibitors (ICIs) exposure, suggesting the presence of independent autoimmune mechanisms.

Fulminant myocarditis (FM) is one of the most severe cardiac complications in MG patients, with its pathogenic mechanisms possibly related to myocardial damage mediated by autoantibodies such as antistriational autoantibodies or systemic inflammatory responses (7–9). Studies on ICI-associated myocarditis have shown that the mortality rate is significantly increased when FM coexists with MG (9, 10), but research on non-ICI-induced MG-FM comorbidity is limited. Existing literature indicates that risk factors for myocarditis in MG patients include thymoma and positive anti-skeletal muscle antibodies (10, 11), but the mechanism underlying FM occurrence in MG patients without thymoma still requires further exploration.

Currently, there are few reports on the coexistence of MG-IM-FM, with most related etiologies attributed to thymoma and/or the use of ICI (12–15). There is a lack of unified guidelines for treatment. The standard regimen includes corticosteroids combined with immunomodulatory therapy, such as intravenous immunoglobulin or plasma exchange, but efficacy is limited in some patients. Efgartigimod, a novel FcRn antagonist, significantly reduces pathogenic antibody levels by accelerating IgG degradation (16) and has been approved for the treatment of generalized MG(gMG). However, its application in the context of MG-IM-FM coexistence remains unreported, particularly regarding its mechanism of efficacy in cardiac function recovery.

This case describes a patient with MG-IM-FM coexistence who had no history of thymoma or ICI exposure. After treatment with efgartigimod combined with intravenous methylprednisolone, the patient’s symptoms and cardiac function were fully restored, and the condition remained stable during a 9-month follow-up.

## Case presentation

A 74-year-old female with a more than 30-year history of coronary heart disease, intermittent oral administration of Suxiao Jiuxin Pills, stent implantation five years ago due to myocardial infarction with normal myocardial enzymes after surgery, and a two-year history of heart failure with previously normal ejection fraction (EF) on echocardiography, and no family history of neurological diseases, was admitted to the hospital because of dropped head syndrome and weakness of all four limbs for 22 days, chest tightness and shortness of breath for 20 days, and dysarthria for 8 days. She was independent in activities of daily living before this admission. On the morning of July 12, 2024, she developed neck extensor weakness, bilateral shoulder weakness, and reduced lingual mobility, without identifiable precipitating factors such as infection, vaccination, or recent medication use. In the afternoon, ptosis of the eyelids and weakness in all four limbs occurred, with symptoms progressively worsening and not alleviated by rest. On July 14, 2024, chest tightness and shortness of breath appeared. Blood tests revealed Aspartate aminotransferase(AST) at 139.7 U/L, Alanine aminotransferase(ALT) at 79.4 U/L, Creatine kinase(CK) at 2855 U/L, and Creatine kinase isoenzyme(CKMB) at 144 ng/ml, all above normal levels. Chest CT scan showed interstitial changes in the lungs. Electrocardiogram: Sinus arrhythmia, first-degree atrioventricular block, intraventricular conduction block, possible inferior wall myocardial infarction, prolonged QT interval. Head MRI showed no new cerebral infarction. The initial diagnosis was not provided by the attending physician, and the patient was only prescribed oral bicyclol. Subsequently, the symptoms worsened. On July 26, 2024, intermittent speech difficulty, masticatory weakness and dysphagia, and dysphagia with aspiration of thin liquids appeared. Ambulatory electrocardiogram findings: sinus rhythm, occasional atrial premature beats, ventricular premature beats some in couplets, complete left bundle branch block, ST-T changes, decreased heart rate variability, echocardiography showing diffuse reduction in wall motion, EF 36%, external hospital considered heart failure, given oral enteric-coated aspirin tablets, bisoprolol fumarate tablets, sacubitril valsartan sodium tablets, furosemide tablets, sustained-release trimetazidine hydrochloride tablets, no improvement. No statins were taken. Due to ptosis, generalized weakness, dysarthria, masticatory weakness and dysphagia, dysphagia with aspiration of thin liquids, shortness of breath, visited our hospital on August 3, 2024.

Physical examination findings: Normal skin, no rashes observed. The palpebral fissure height was 5 mm and marginal reflex distance1(MRD1) was 2.0 mm bilaterally, significantly lower than the normal range, unable to look up, lagophthalmos, no diplopia. Facial weakness with air leakage on cheek puffing. Fatigue when chewing soft food, dysphagia, inability to eat, dysarthria, dyspnea in supine position. Neck muscle strength grade 1, proximal muscle strength of the limbs grade 2, distal muscle strength grade 3 according to the Medical Research Council (MRC) grading scale, unable to maintain the arms in a horizontal position or elevate the legs to 45 degrees.

**Figure 1:**
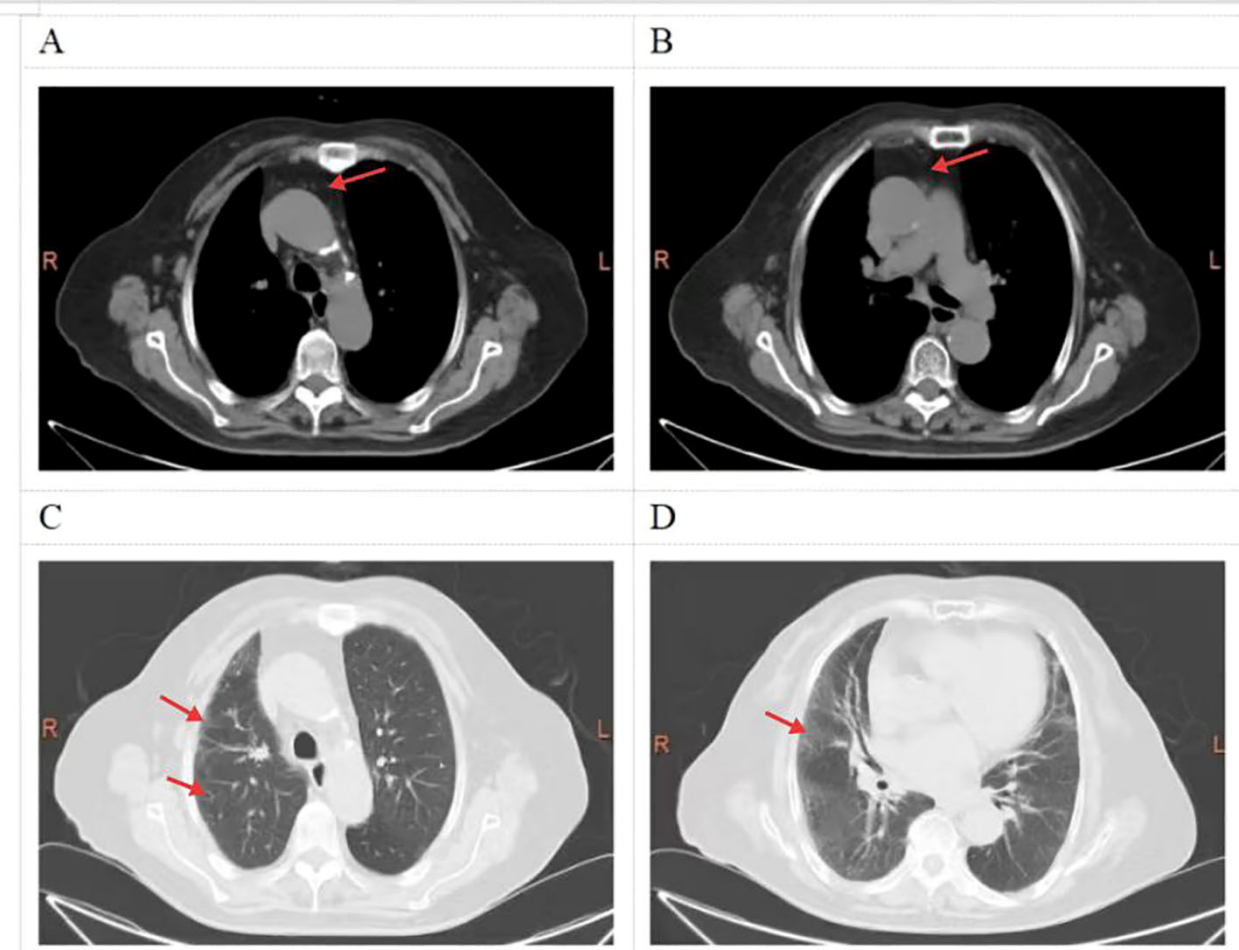
Chest CT scan. **(A, B)** show no space-occupying lesions in the anterior mediastinum or lungs; **(C, D)** demonstrate bronchiectasis and interstitial changes in the lungs.

**Figure 2:**
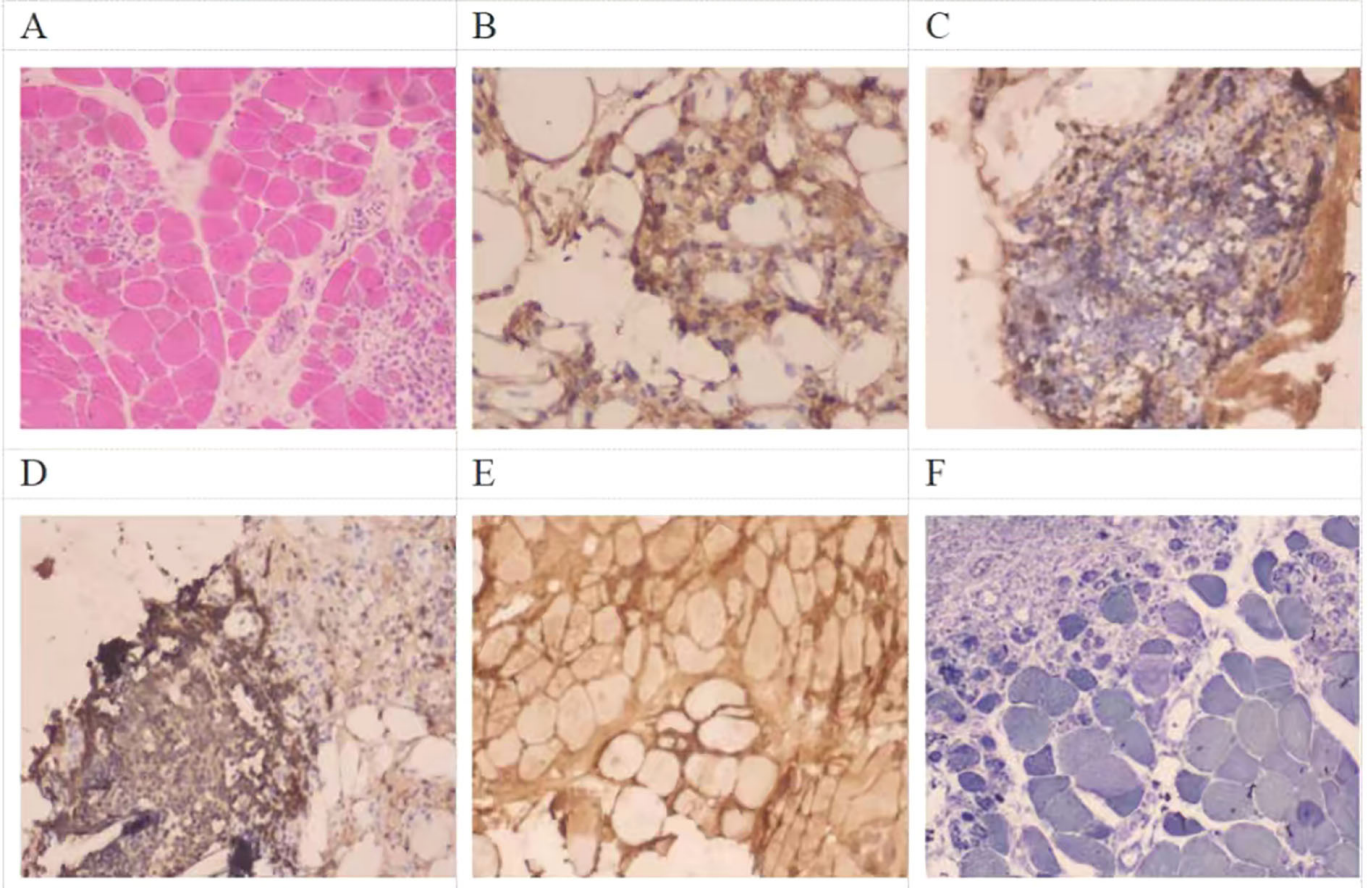
Pathological Findings of Biceps Brachii Muscle Biopsy **(A)** Hematoxylin and eosin (HE) staining; **(B)** CD4-positive T lymphocyte infiltration; **(C)** CD8-positive T lymphocyte infiltration; **(D)** CD20-positive B lymphocyte infiltration; **(E)** Sarcolemmal MHC-I expression; **(F)** Abnormal staining on NADH dehydrogenase staining.

Testing results: Acetylcholine receptor antibody(AchR-Ab) 3.65 nmol/L positive (radioimmunoassay), MUSK, LRP4, Titin, RyR, SOX1, Agrin antibodies all negative; ALT 111 U/L(7-40U/L), AST 147 U/L(13-35U/L), CK 3144 U/L(40-200U/L), CKMB 204.99 ng/mL(0-5ng/ml), Albumin(ALB) 33.0 g/L(40-55U/L); Myoglobin(Myo) 2972.5 ng/mL(14.3-65.8ng/mL), High-sensitivity troponin I(hs-cTnI) 0.087ng/mL (0-0.0116ng/mL); Antinuclear antibody spectrum(0-20): Anti-Jo-1 antibody 88.48 AU positive, Anti-Ro-52 antibody 55.20 AU positive, Anti-dsDNA antibody 48.48 IU/mL positive, Anti-nRNP antibody 152.66 AU positive, Anti-nuclear antibody positive: cytoplasmic granular pattern (1:320) and nuclear granular pattern (1:3200), detected by indirect immunofluorescence (IIF) assay; B-type natriuretic peptide 114 pg/mL; CT shows: Bilateral bronchiectasis with infection; Partial interstitial changes; No mass detected in the premediastinum or lungs (**Figure 1**). Electromyography: Repetitive nerve stimulation of the bilateral deltoid muscle demonstrated a decremental response at low frequencies without an incremental response at high frequencies. Repetitive nerve stimulation of the bilateral facial, median, and common peroneal nerves showed no abnormal responses. Needle electromyography revealed spontaneous potentials. Echocardiogram and cardiac function measurement: General reduction in left ventricular wall motion, enlarged left atrium, calcification of the aortic valve, mild mitral regurgitation, reduced left ventricular systolic function, heart function EF 18%. Electrocardiogram shows: Sinus rhythm, Ventricular premature beats, Complete left bundle branch block, QS pattern in lead III AVF. Due to extremely poor cardiac function, neostigmine test was not performed. Right biceps brachii biopsy: HE staining shows involvement of all muscle fibers microscopically. Muscle fibers exhibit marked heterogeneity in size. Extensive focal inflammatory cell infiltration is observed between muscle fibers and around blood vessels. The interstitium is markedly edematous. T lymphocyte infiltration, primarily positive for CD8, with visible B lymphocyte infiltration positive for CD20. The sarcolemma shows positive expression of MHC-1 staining. (**Figure 2**).

The patient was diagnosed with: 1. Myasthenia gravis(MGFA IVb) 2. Inflammatory myopathy(IM) 3. Fulminant myocarditis(FM) 4. Connective tissue disease 5. Interstitial lung disease 6. Bronchiectasis with infection 7. Hypoproteinemia 8. Coronary artery atherosclerotic heart disease 1) Old myocardial infarction 2) Post-coronary artery stent placement.

**Figure 3:**
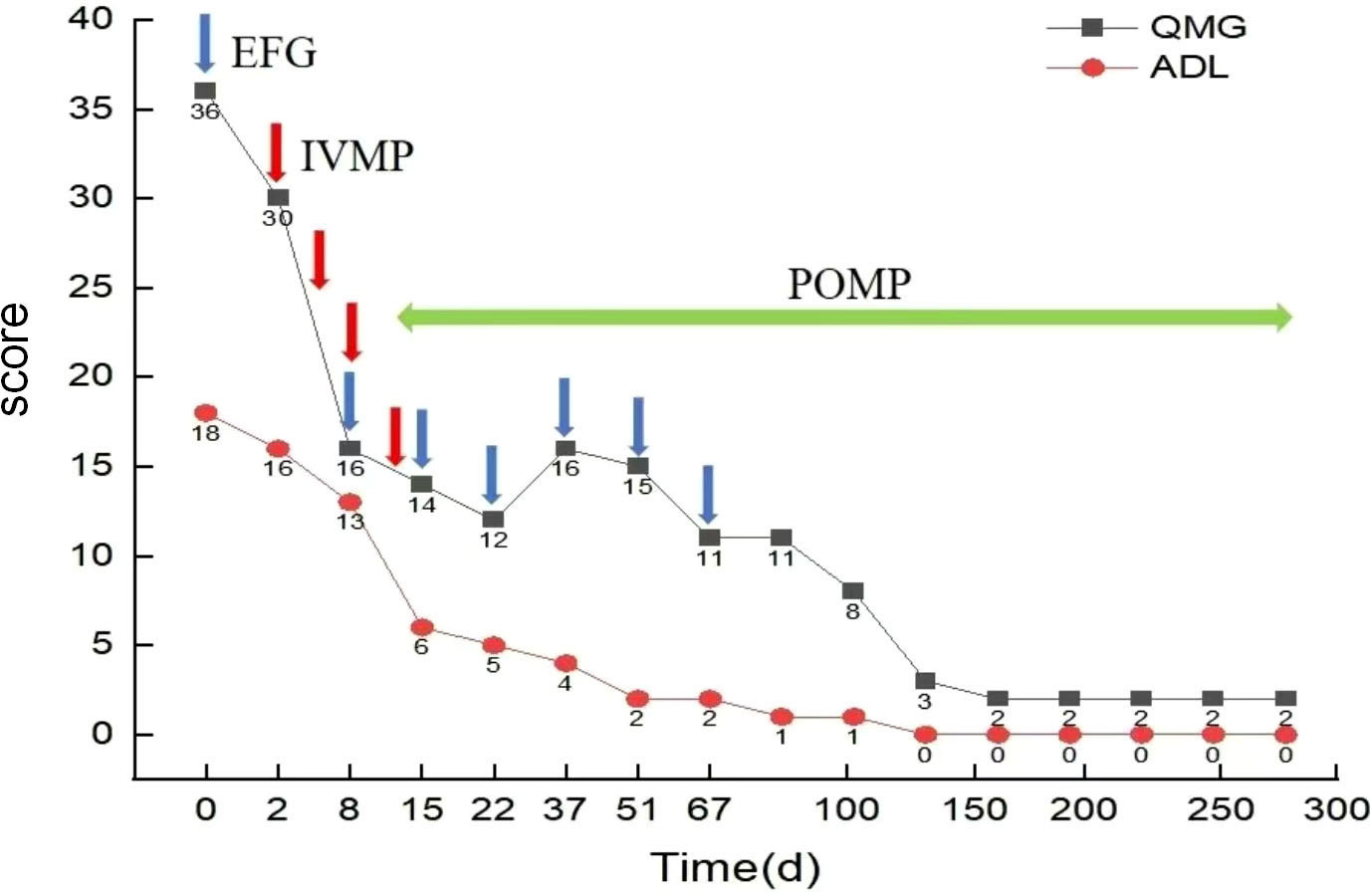
Medication Regimen and Score Changes. The figure shows the administration timeline of efgartigimod combined with methylprednisolone, as well as changes in QMG and MG-ADL scores. EFG: efgartigimod; IVMP: intravenous methylprednisolone; POMP: per oral methylprednisolone.

Treatment: After admission, a nasogastric tube was placed for enteral nutrition support. On August 6, 2024, 600 mg of efgartigimod was administered intravenously. By August 8, 2024, ptosis of the eyelids and dysarthria had improved, and re-examination of CK, CKMB and MYO levels showed a decrease. Intravenous administration of methylprednisolone at a dose of 0.25 g was initiated on the same day (August 8, 2024) for hormone pulse therapy, reducing by three days until switching to oral prednisone 60mg, combined with weekly intravenous administration of 600 mg of efgartigimod for four weeks, then switch to 600 mg intravenous administration every 2 weeks. (**Figure 3**).

**Figure 4:**
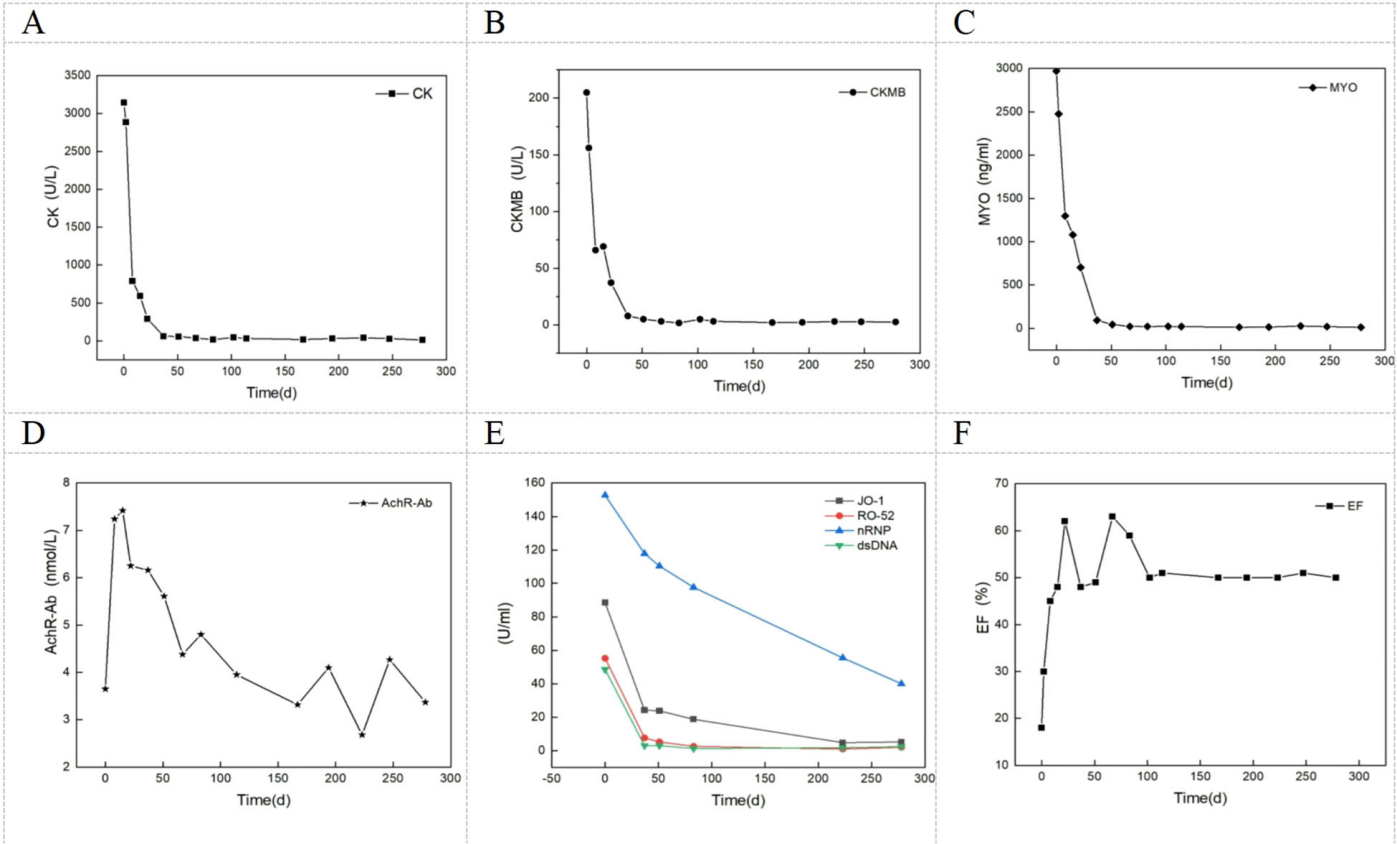
Abnormal Indicator Changes and Core Variation Rules (Baseline to 9-Month Follow-Up) **(A–C)** CK, CKMB, MYO returned to normal within 40-50 days; **(D)** AchR-Ab decreased approximately 50%; **(E)** anti-JO-1, RO-52, dsRNA-Ab returned to normal; anti-nRNP-Ab decreased to twice the normal value; **(F)** LVEF returned to normal around day 70 and remained stable.

Treatment outcome: The patient’s muscle weakness symptoms gradually improved. On August 17, 2024, the nasogastric tube was removed, and oral feeding proceeded smoothly. Neck muscle strength was grade 4+, proximal limb muscle strength was grade 2, and distal limb muscle strength was grade 3 according to the Medical Research Council (MRC) grading scale. Scores for MG-QMG and MG-ADL decreased (**Figure 3**). Re-examination showed that CK, CKMB and MYO levels returned to normal. Echocardiography indicated a gradual increase in LVEF to normal levels. AchR-Ab levels have decreased by nearly half from their peak. Antibodies against Jo-1, Ro-52, and dsDNA all normalized, while the anti-nRNP decreased to twice the normal value (**Figure 4**). After 9 months of follow-up, the patient exhibited normal eating, speech, and breathing abilities, could walk independently, and maintained stable muscle weakness symptoms with normal enzyme markers and normal LVEF. Currently, the patient is taking 15 mg of prednisone daily and 50 mg of cyclophosphamide daily, with doses being reduced according to plan.

## Discussion

This case report describes a rare patient with a coexistence of MG, inflammatory myopathy, and fulminant myocarditis, not associated with thymoma or exposure to ICIs. The patient’s characteristics include: non-fluctuating muscle weakness without myalgia, widespread muscle involvement including extraocular muscles. Elevated muscle enzymes were observed, along with positivity for AchR-Ab, antinuclear antibodies, Jo-1-Ab, RO-52-Ab, nRNP-Ab, among others. Electromyography showed low-frequency decrement on repetitive stimulation, and needle electromyography revealed spontaneous potentials. The patient also presented with pulmonary interstitial lesions, arrhythmias, heart failure with an EF of 18%, and no thymic abnormalities or history of ICIs exposure. Unfortunately, due to shortness of breath, the patient could not undergo cardiac and muscle MRI. Treatment with efgartigimod combined with intravenous methylprednisolone resulted in improvement of neuromuscular symptoms, normalization of kinase levels, and significant recovery of cardiac function, with EF improving from 18% to over 50%.

This case presented atypical clinical features consistent with immune-related multi-system involvement reported in literature, but with unique pathogenesis. MG concurrent with myositis is uncommon, often accompanied by thymic pathology and anti-AChR antibody positivity (13, 17). In this patient, AchR-Ab were positive, but no thymic lesions were observed. Literature reports that myocardium and skeletal muscle can be autoimmune targets in MG patients (10), leading to cardiomyopathy, myocarditis, and arrhythmias; however, MG concurrent with myocarditis, especially fulminant myocarditis, is relatively rare (18, 19). Thymoma and history of myasthenic crisis (MC) are risk factors for myocarditis in MG patients. Antistriational autoantibodies (such as anti-titin antibodies) have been associated with MG combined with myocarditis in literature, but such antibodies were not detected in this case, suggesting the possibility of other unknown autoantibody targets. Inflammatory myopathies involving myocardium are typically presented through case reports, case series, and cohort studies, without reaching conclusions that alter clinical practice. Some reports describe fulminant myocarditis with poor prognosis (20). The presence of Jo-1 antibodies in this case suggests that inflammatory myopathy may cross-react with myocarditis via molecular mimicry mechanisms (21). ICIs can induce a triad of MG, myositis, and myocarditis (7, 19), but this case suggests that non-ICI-induced autoimmune disorders can also lead to similar overlapping syndromes. The presence of multiple autoantibodies indicates that a potential connective tissue disease may have driven multisystem immune attacks.

Glucocorticoids are first-line drugs for treating autoimmune diseases such as MG, myositis, and fulminant myocarditis, but they may cause transient exacerbation of severe MG during treatment (22). Efgartigimod targets FcRn to reduce pathogenic IgG levels (23–26), showing rapid efficacy and was approved by the National Medical Products Administration (NMPA) of China on June 30, 2023, for use in combination with conventional therapy in adult patients with AchR-Ab positive gMG, with potential adverse reactions including infections and hypersensitivity reactions. It has also been reported for use in neuromyelitis optica, chronic inflammatory demyelinating polyradiculoneuropathy, Guillain-Barré syndrome, and stiff person syndrome (27–32). There are no reports on polymyositis or fulminant myocarditis, but given that this patient has multiple positive autoantibodies, we reasonably infer that using efgartigimod to clear anti-Jo-1 antibodies, anti-Ro-52 antibodies, anti-dsDNA antibodies, and anti-nRNP antibodies has significant therapeutic potential. We initially administered 600 mg of efgartigimod intravenously, and by the next day, the ptosis of both eyelids and dysarthria had improved. By the third day, creatine kinase and myoglobin levels had decreased. Considering the patient’s economic situation and the complexity of the disease, we combined efgartigimod with intravenous methylprednisolone.

The mortality rate is significantly increased in patients with FM combined with severe MG (19). FM often requires mechanical circulatory support, such as ECMO (33). This case demonstrates that early combined pharmacotherapy can reverse cardiac function, suggesting that early immunomodulation may avoid invasive treatments. Efgartigimod successfully reduces human serum IgG levels, which may be crucial for rapidly improving neuromuscular symptoms and myocarditis. On June 30, 2023, the National Medical Products Administration (NMPA) of China approved its indication in combination with conventional therapy for the treatment of adult patients with generalized MG who are positive for AchR-Ab. Potential adverse reactions include infections and hypersensitivity reactions. However, its effects on myocarditis and myopathy remain unclear and require further data. The combination of efgartigimod and glucocorticoids shows synergistic effects; literature suggests it can reduce steroid dosage (34, 35). In this case, the reduction of steroids after steroid pulse therapy proceeded smoothly, and the absence of disease rebound during the process further supports this point. Adverse events reported for efgartigimod include mild headache, fatigue, and back pain, with no occurrences of allergy or severe events (24, 25). No infections or albumin decline were observed in this case.

Clinical Implications and Limitations: This case represents the first reported successful application of efgartigimod in the coexistence of MG, inflammatory myopathy, and fulminant myocarditis, thereby expanding its therapeutic spectrum. However, several considerations remain: the precise effect of efgartigimod on non-AChR-Ab, such as Jo-1, requires further investigation; corticosteroids remain the first-line treatment for MG, inflammatory myopathy, and fulminant myocarditis, and the synergistic effect of efgartigimod combined with intravenous methylprednisolone needs confirmation through controlled trials involving placebo or standard therapy groups; additionally, future research should identify common antigen targets and optimal treatment combinations for such multisystem autoimmune diseases. In this case, no thymic abnormalities or tumors were observed, but vigilance is required for the possibility of immune dysregulation preceding tumor development, necessitating screening for tumors during subsequent follow-ups.

## Conclusion

This case expands the clinical spectrum of MG overlap syndrome, confirming that autoimmune disorders without typical triggers can lead to a life-threatening clinical combination of MG, IM and FM. This condition typically presents acutely, progresses rapidly, and is critically severe; clinicians should remain highly vigilant, as early intervention is crucial for improving prognosis. The immunological mechanisms underlying its occurrence remain unclear and require further investigation. Conventional glucocorticoids still serve as first-line treatment, but the novel targeted drug efgartigimod, which has fewer adverse effects and faster onset, may offer a promising alternative, though larger sample sizes are needed for validation.

## Data Availability

The original contributions presented in the study are included in the article/supplementary material. Further inquiries can be directed to the corresponding author.
